# Sex chromosomes in meiotic, hemiclonal, clonal and polyploid hybrid vertebrates: along the ‘extended speciation continuum'

**DOI:** 10.1098/rstb.2020.0103

**Published:** 2021-09-13

**Authors:** Matthias Stöck, Dmitrij Dedukh, Radka Reifová, Dunja K. Lamatsch, Zuzana Starostová, Karel Janko

**Affiliations:** ^1^ Leibniz-Institute of Freshwater Ecology and Inland Fisheries - IGB (Forschungsverbund Berlin), Müggelseedamm 301, 12587 Berlin, Germany; ^2^ Amphibian Research Center, Hiroshima University, Higashi-Hiroshima 739-8526, Japan; ^3^ Institute of Animal Physiology and Genetics, Laboratory of Fish Genetics, The Czech Academy of Sciences, 277 21 Libechov, Czech Republic; ^4^ Department of Zoology, Faculty of Science, Charles University, Viničná 7, Prague 2, 128 00, Czech Republic; ^5^ Research Department for Limnology, University of Innsbruck, Mondseestrasse 9, A-5310 Mondsee, Austria; ^6^ Department of Biology and Ecology, Faculty of Science, University of Ostrava, 701 03 Ostrava, Czech Republic

**Keywords:** sex chromosomes, hybridization, evolution, clonal reproduction, speciation

## Abstract

We review knowledge about the roles of sex chromosomes in vertebrate hybridization and speciation, exploring a gradient of divergences with increasing reproductive isolation (speciation continuum). Under early divergence, well-differentiated sex chromosomes in meiotic hybrids may cause Haldane-effects and introgress less easily than autosomes. Undifferentiated sex chromosomes are more susceptible to introgression and form multiple (or new) sex chromosome systems with hardly predictable dominance hierarchies. Under increased divergence, most vertebrates reach complete intrinsic reproductive isolation. Slightly earlier, some hybrids (linked in ‘the extended speciation continuum') exhibit aberrant gametogenesis, leading towards female clonality. This facilitates the evolution of various allodiploid and allopolyploid clonal (‘asexual’) hybrid vertebrates, where ‘asexuality' might be a form of intrinsic reproductive isolation. A comprehensive list of ‘asexual' hybrid vertebrates shows that they all evolved from parents with divergences that were greater than at the intraspecific level (K2P-distances of greater than 5–22% based on mtDNA). These ‘asexual' taxa inherited genetic sex determination by mostly undifferentiated sex chromosomes. Among the few known sex-determining systems in hybrid ‘asexuals', female heterogamety (ZW) occurred about twice as often as male heterogamety (XY). We hypothesize that pre-/meiotic aberrations in all-female ZW-hybrids present Haldane-effects promoting their evolution. Understanding the preconditions to produce various clonal or meiotic allopolyploids appears crucial for insights into the evolution of sex, ‘asexuality' and polyploidy.

This article is part of the theme issue ‘Challenging the paradigm in sex chromosome evolution: empirical and theoretical insights with a focus on vertebrates (Part II)’.

## 1. Introduction

Our understanding of speciation has evolved from being regarded as a long and steady process, governed by natural selection in various forms [1–3], to a view that includes dynamic and/or reticulate and potentially fast processes [4–9]. Speciation may occur in parallel under similar ecological conditions [10]. In allopatry, incipient species accumulate subtle differences along the entire genome [11,12] with single speciation genes [13] being the first witnesses and perhaps sometimes the drivers of speciation.

**Figure 1.**
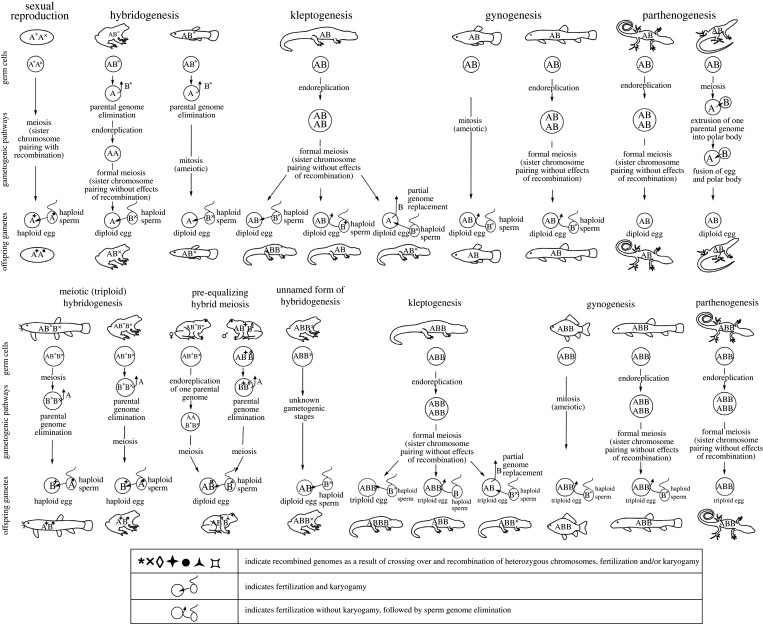
Clonal, hemiclonal and meroclonal reproductive modes of hybrid vertebrates (diploids: upper row; triploids: lower row) in comparison with sexual reproduction (upper left). Each column shows parental individuals, gametogenic pathways with germ cells, gametes and offspring genome composition (expanded from Lamatsch & Stöck [49] and Stöck *et al.* [50]. **Letters and symbols**: A, B: genomes of parental species that formed the hybrid taxon/form; when lacking additional modifying symbols, the genomes are usually inherited and transmitted clonally; symbols are explained overleaf, below the figure. **Description of reproductive modes: Sexual reproduction** (upper row): oogonia enter a normal meiosis, which results in recombined haploid ova; after fertilization by haploid sperm from conspecific males, diploid offspring with recombined maternal and paternal genomes form diploid male or female offspring. **Hybridogenesis** (upper row): hemiclonal reproductive mode, during which the genome of one parental species is eliminated from the germ cells [51–56]; the genome of the other parental species is either endoreplicated and undergoes meiosis without effects of recombination (e.g. diploid water frog, *Pelophylax esculentus* [55,57,58]) or gametogenesis is ameiotic (e.g. the livebearing fish *Poeciliopsis monacha-lucida* [51,56]). Diploid hybrid offspring emerge after fertilization of the haploid ovum by recombined allospecific sperm, usually from a parental, sexual species. **Unnamed form of hybridogenesis** (lower row): Clonal diploid eggs are possibly formed by the elimination of one of the double copied genomes while the remaining genomes undergo endoreplication followed by meiosis without effects of recombination (*P. esculentus* example [59,60]). **Kleptogenesis:** (upper and lower rows): occurs in unisexual salamanders, *Ambystoma* [61]. The genome of germ cells is endoreplicated, undergoes meiosis without effects of recombination resulting in diploid eggs (above) or triploid eggs (below) [62,63]. Ova may either be activated by allospecific sperm without karyogamy, i.e. like in gynogenesis (middle), be truly fertilized, leading to ploidy elevation of offspring (left), or sperm may in part replace one of the maternal genomes in the egg, followed by its partial elimination (right) [54]. **Gynogenesis** (upper and lower rows): formation of clonal gametes by an ameiotic process (example: *Poecilia formosa*, upper row [47,64]; example: *Carassius langsdorfii*, lower row [65]) or endoreplication (example: diploid *Cobitis elongatoides-taenia*, upper row [66,67]; example: triploid *Cobitis 1elongatoides-2taenia*, lower row [66–68]) of genomes in germ cells followed by meiosis without effects of recombination. Diploid gametes (upper row) or triploid gametes (lower row) are fertilized without karyogamy, followed by sperm genome elimination. **Parthenogenesis** (upper and lower row): Clonal gametes form via endoreplication of genomes in germ cells followed by meiosis without effects of recombination (example: *Aspidoscelis tesselatus,* upper row [69]; example: *Aspidoscelis uniparens*, lower row [70]). Alternatively, the genome of one parental species is extruded into the polar body and then fuses with the egg, restoring diploidy (*Darevskia unisexualis*) [71]. Eggs develop without sperm/fertilization. **Meiotic (triploid) hybridogenesis** (lower row): recombined haploid gamete formation after meiotic (example: *Misgurnus anguillicaudatus* [72]) or premeiotic elimination of a single copied genome (example: *P. esculentus* [55]); offspring are diploid. **Pre-equalizing hybrid meiosis** (lower row): occurs in allotriploid Batura toads (*Bufo(tes) baturae*) [50,73] and presumably also in related taxa (§5c(iii)). In females (left), a single copy genome (A) is separately endoreplicated and enters meiosis as ‘pseudo-bivalents' (box 1), along with bivalents of heterozygous chromosomes from another parental species (BB′). The formally tetraploid meiosis results in diploid gametes. Males (right) eliminate the single-copy clonal genome (A), while the two remaining genomes (B) undergo a normal meiosis (BB′) and form haploid recombined sperm. Batura toads present the only known gonochoristic vertebrate taxon with simultaneous Mendelian (BB′) and clonal (A) genome transmission. Fertilization results in triploid offspring of both sexes.

In this paper, after a lead-in on intrinsic reproductive isolation and on sex chromosomes in speciation, we explore a gradient of divergences (the ‘speciation continuum' [14], detailed below) to review knowledge about the evolutionary impact of sex chromosomes under hybridization in vertebrates. We start our ‘evolutionary journey’ through speciation from the early onset of evolutionary divergence in near-panmictic populations that form meiotic hybrids. We then examine sex chromosomes by moving along various stages of increasing divergences and accumulating intrinsic reproductive isolation between hybridizing species (table 1) until a stage is reached, when hybrid vertebrates evolve that rarely exhibit so-called ‘asexual' (some forms of hybrid clonal and allopolyploid) reproductive modes (box 1 and figure 1). Our way of studying and thinking about sex chromosomes in (mostly allopatric) speciation may offer a useful framework (table 1). We discuss the current state of the field, focusing on available knowledge and major research gaps on sex chromosomes in various kinds of vertebrate hybrids.

**Table 1.** Hypothetical evolutionary stages with empirical examples along the ‘extended speciation continuum', with effects under secondary contact and hybridization, with special attention on undifferentiated and differentiated sex chromosomes. This table is supposed to show evolutionary tendencies as described in the text. Note that stages along the ‘extended speciation continuum' do not necessarily correspond to absolute divergence times as in some species, speciation proceeds more rapidly than in others, i.e. stages should be preferentially compared within a certain radiation of organisms. Evidence for *Aspidoscelis* lizards is equivocal since sex chromosomes are only known in very few species.

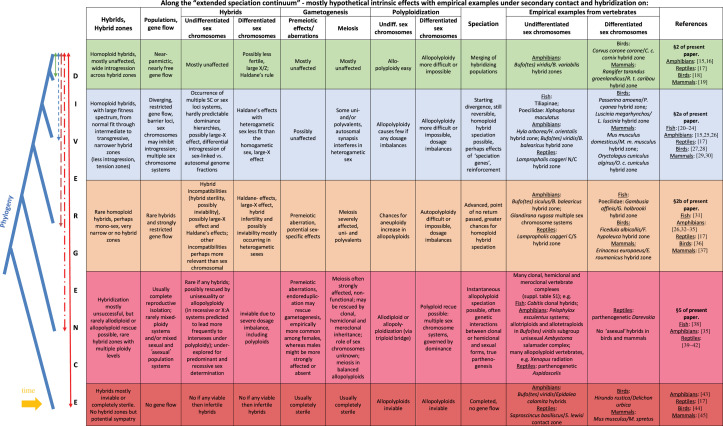

### (a) The evolution of intrinsic reproductive isolation

Intrinsic postzygotic isolation (i.e. decreased fertility, sterility or even inviability of interspecific hybrids) is an important spectrum of mechanisms of reproductive isolation that prevents many related species from merging [4]. For more than 80 years, there has been a prevailing view that intrinsic postzygotic isolation arises as a result of accumulating (Bateson–)Dobzhansky–Muller (BDM) incompatibilities at individual genes that diverged between species to a degree preventing proper chromosome pairing or interaction of their protein products in hybrids [79–81]. The search for ‘speciation genes' involved in such incompatibilities led to the discovery of several candidates in various taxa [82,83]. Such candidate genes have common characteristics, defined by relatively fast evolution, often driven by positive selection and coevolutionary arms races (e.g. [84–86]). Nevertheless, the evolution of intrinsic postzygotic isolation is a complex process that, beyond incompatibilities between individual protein products as assumed by the original Dobzhansky–Muller model, includes additional mechanisms. For instance, it may be driven by overall divergence of noncoding DNA [87], as similarly predicted by Bateson [88], whose concept is analogous to a current chromosomal speciation model [89]. It predicts diverging lineages to accumulate mutually incompatible changes in karyotypes, causing problems in meiotic homology search, synapses and bivalent formation in hybrids, leading to aborted gametogenesis [90,91]. Reproductive isolation may also result from a disrupted regulatory cross-talk between merged genomes [92], which may, for example, result in the activation of transposable elements in hybrid genomes [93–96].

Box 1. **Glossary (definitions in part after Avise [46]).**

**Allospecific** (=heterospecific): belonging to different taxonomic species.

**Asexual reproduction:** *sensu stricto*: Any form of reproduction that does not involve the fusion of sex cells (gametes); i.e. a reproductive mode, by which an organism passes on its genome clonally by circumventing the effects of recombination and meiotic reduction during gametogenesis; therefore, the genome is transmitted unaltered. This is achieved by different mechanisms. Some organisms transmit their genomes strictly asexually, i.e. in a completely *clonal* way (*parthenogenesis*, see below). In this paper, when we write ‘asexual’ (i.e. in quotation marks), we use the term *sensu lato:* some organisms transmit only parts of their genomes clonally, while the rest is eliminated and replaced in each generation by a sexually reproducing parental species (sexual host) (*hybridogenesis*). Many such organisms show a strong female bias (see: *unisexual species*). The literature uses the terms *asexual* and *asexuality* sometimes uncritically, causing scientific disputes over ‘asexual’ organisms, their evolution and long-term survival. Different mechanisms also exist with respect to the requirement (or not) for fertilization. True parthenogens are completely independent of sperm (and thus of males), while other types of ‘asexuals’, *gynogens* or *sperm-dependent parthenogens*, rely on insemination, usually, but not always, from closely related sexual species [47]. The sperm either only triggers embryogenesis while its genome gets eliminated after fertilization (*gynogenesis*, pseudogamy) (but it may also contribute genetically to the progeny either by subgenomic amounts, such as microchromosomes [48]), or the entire sperm genome may be incorporated into the progeny, resulting in ploidy elevation (genome addition); or elimination, after one generation—in the next round of gamete production (in some forms of *hybridogenesis*). Subgenomic amounts of sperm-DNA can occasionally also be incorporated into the egg and partly replace or perhaps recombine with the maternal genome (*kleptogenesis*); the paternal incorporation may serve to ‘purge’ deleterious mutations. See figure 1 for ‘asexual’ (*sensu lato*) reproductive modes in vertebrates.

**Automixis:** form of ‘asexual reproduction’ that includes the union of meiotic products of an individual (note: some authors use the term more broadly to encompass any form of uni-individual reproduction that includes meiosis or a meiosis-type process, including premeiotic endomitosis).

**Bisexual:** a population or species composed of male and female (=*gonochoristic*) individuals.

**Clone:** (*noun*) biological entity (e.g. gene, cell, or multicellular organism) that is genetically identical to another; alternatively, all genetically identical entities that have descended ‘asexually’ from a given ancestral entity; (*verb*) to produce such genetically identical entities or lineages.

**Clonal**: mode of inheritance by which the entire genome is transmitted unaltered (although rarely subgenomic amounts of DNA may be added or altered).

**Conspecific:** belonging to the same taxonomic species (opponyms: allospecific, heterospecific).

**Premeiotic endoreplication (=endomitosis**): chromosomal replication within a cell that does not divide.

**Gamete**: a mature reproductive cell (egg or sperm).

**Gametogenesis:** the process by which sex cells are produced.

**Germline:** the lineage of cells leading to an individual's *gametes*.

**Gynogenesis** (synonym: sperm-dependent parthenogenesis or pseudogamy): see figure 1.

**Hemiclone:** the portion (classically 50%) of a genome that is transmitted intact, without recombination in a hybridogenetic lineage.

**Hemiclonal reproduction**: mode of inheritance by which gamete production is partly (classically 50%) clonal, like in diploid *hybridogenesis*.

**Heterogametic sex:** the sex that produces gametes that each contain one of two different types of sex chromosomes.

**Heterozygosity:** the percentage of heterozygotes or loci in a heterozygous state in an organism or population.

**Heterozygotes:** a diploid organism possessing two different alleles at a specified genetic locus.

**Homozygotes:** a diploid organism possessing the same alleles at a specified genetic locus.

**Homogametic sex:** the sex that produces gametes that all contain the same type of sex chromosomes.

**Hybridization:** the successful mating of individuals belonging to genetically different populations, lineages, or species.

**Hybridogenesis:** see figure 1.

**Intrinsic reproductive isolation**: genetically caused post-zygotic mechanisms such as hybrid inviability, decreased fertility, sterility and hybrid breakdown that prevent sexual organisms from producing fully fertile multi-generation hybrids.

**Introgression:** the movement of genes (gene flow) between populations, lineages, or species via hybridization.

**Kleptogenesis:** see *asexual reproduction* and figure 1.

**K2P-corrected distances**: nucleotide-sequence divergences (here based on mitochondrial DNA) calculated using the Kimura-two-parameter (K2P) model, the best metric when genetic distances are low [74].

**Meiosis:** the cellular process whereby a germline cell divides to form gametes containing half the chromosomes of the parent cells (usually including crossing over and recombination).

**Meroclonal:** (*mero-*, Greek: ‘partial’) partly clonal gamete production of triploid (or other polyploid) organisms, first described from allotriploid water frogs.

**Mitosis:** the process of cell division that produces daughter cells with the same chromosomal constitution as the parental cells.

**Oogenesis:** the production of oocytes, egg cells or ova.

**Parthenogenesis:** see also *asexual reproduction* and figure 1; *obligate parthenogenesis* is a reproductive mode by which offspring (at least an embryo) is produced from an egg without genetic contribution of sperm; in vertebrates, this reproductive mode is mostly of hybrid origin, but see [75] for potential exceptions; some non-hybrid vertebrate clades (sharks, reptiles) can reproduce (occasionally) by so-called *facultative parthenogenesis* [76–78], which is neither of hybrid origin nor in the focus of this paper.

**Paternal leakage:** the occasional incorporation of a sperm or its mtDNA into an ovum of a gynogenetic organism and thereby into the resulting offspring.

**Pseudo-bivalent:** bivalent containing two identical (homozygous) chromosomes as a result of premeiotic endoreplication.

**Sexual reproduction:** prevailing mode of reproduction in metazoans, characterized by production of offspring via syngamy of meiotically produced gametes. Recombination and segregation of chromosomes (alleles) during meiosis result in genetically variable gametes and offspring.

**Unisexual species:** a species consisting exclusively of females or sometimes also applied to species with a strong female-bias.

### (b) The prominent role of sex chromosomes in speciation

Sex chromosomes play key roles at the origin of intrinsic postzygotic reproductive isolation [97–99]. Research in many animals, including vertebrates, led to two more or less general ‘rules of speciation' involving sex chromosomes: (i) Haldane's rule, predicting increased sterility or inviability of the heterogametic sex (i.e. XY males or ZW females) [100,101] and (ii) the large-X effect ([102]; discussed in [103,104], assuming a disproportionately large effect of the X chromosome (or the Z chromosome in organisms with heterogametic females) on reduced hybrid fitness compared to autosomes. Both rules were generally attributed to recessive hybrid incompatibilities, manifested if present on the hemizygous parts of the X or Z chromosomes in the heterogametic sex. In addition, such incompatibility loci may be manifested if present on the non-pairing Y or W chromosomes—these, however, usually harbour relatively few genes and their role for speciation may thus be limited, even in strongly heteromorphic sex chromosomes like in mammals and birds [81].

Other explanations of Haldane's rule and the large X-effect may include generally faster rates of molecular evolution on the X and Z chromosomes [105,106], rapid coevolutionary arms races between sex-linked segregation distorters and their suppressors [107] or failure of epigenetic inactivation of sex chromosomes during meiosis [108,109]. A possible activation of endogenous retroviruses on the W chromosome may also explain Haldane's rule in birds with highly heteromorphic sex chromosomes [96]. Filatov ([110] and citations therein) recently concluded that haploid expression and species-specific Y-degeneration need more attention regarding their roles in speciation. Thus, both major rules of speciation may represent composite phenomena, resulting from different causes active in different contexts [111]. Until recently, undifferentiated sex chromosomes have been hardly accessible by genetics for many species, and empirical sex chromosomal sequence data are just becoming available through chromosome-scale genomics.

### (c) The speciation continuum of diploid lineages

Reproductive isolation of diploid lineages tends to increase with genetic distance [87,112], and thus with divergence time [15,17,113], usually as a series of ‘small steps rather than a single genetic revolution' [114]. In this ‘speciation continuum' [14,115,116], we witness diverging evolutionary lineages anywhere between near-panmictic populations along various levels of partial separation up to complete reproductive isolation, causing many of the controversies over ‘what is a species?' [117,118]. Diverging lineages often show permeable boundaries across some parts of the genome, while loci underlying reproductive isolation resist introgression, resulting in a highly heterogeneous differentiation landscape across the genome. This includes regions with low differentiation as well as genome parts that are considerably differentiated (differentiation islands), potentially corresponding to loci resistant to introgression [119–121]. Proportions of such differentiated regions may expand with divergence time and accumulate reproductive isolation. This also allows measuring the speciation stage for a given pair of species [122] (table 1).

Usually, when the divergence between incipient species increases, so does the amount of incompatibilities, negatively affecting the fitness of interspecific hybrids [11,123,124]. Along this speciation continuum [14], hybrid fitness may in some cases even increase (hybrid vigor), potentially facilitating introgression. Nevertheless, at later stages, hybrids' fitness inevitably decreases (see 3.1), often first being affected by impaired gametogenesis and other adverse effects. These include impairments of the ability to reproduce, often initially affecting the heterogametic hybrids [100,125,126], and subsequently by reaching complete reproductive isolation (complete infertility or inviability of hybrids). This trajectory suggests that pre-meiotic and meiotic gametogenetic processes may be more vulnerable to intergenomic incompatibilities than traits related to the viability of hybrids (see §3a).

## 2. Sex chromosomes in hybrids along the speciation continuum

### (a) Sex chromosomes of hybrids in early stages of divergence: introgression, genetic interaction and/or dominance and multiplication

Under secondary contact of diverging lineages, introgression in hybrid zones into the parental gene pools requires that some of the hybrids are fertile and can backcross with the parental lineages. Multi-generation backcrosses only occur between incipient species, i.e. under incomplete reproductive isolation.

Generally, in such situations, X and Z chromosomes introgress less across the hybrid zones than do autosomes in many vertebrates, including fish [127], birds [27,28,36] and mammals [29,30]. Most of these taxa feature heteromorphic sex chromosomes, suggesting that greater heteromorphy and thus hemizygosity (i.e. unequal gene content causing potential dosage imbalances) increase the chances for sex chromosome dosage imbalances and postzygotic hybrid incompatibilities (Haldane effects). This was also supported by simulations [128]. In fruit flies (*Drosophila*) with large-sized sex chromosomes, intrinsic postzygotic isolation evolved relatively earlier than in species possessing smaller sex chromosomes [129].

So far, only some empirical population genetic studies have been accomplished in hybrid zones with undifferentiated sex chromosomes, comparing introgression at sex-linked versus autosomal markers. Data from amphibians with homomorphic sex chromosomes pointed to large X-effects in hylid frogs [25] or apparent absence of such effects in bufonid toads [26]. A metastudy of interspecies crosses suggested that higher levels of sex chromosome heteromorphism were associated with stronger reproductive isolation [130]. Taken together, among closely related lineages, sex chromosome introgression appears to be easier the less differentiated these sex chromosomes are.

Several examples from teleosts suggest that introgression of sex chromosomes in an early stage of divergence of evolutionary lineages may not only result in interactions among parental sex chromosomes (e.g. in hybrid zones), but even in the evolution of multiple sex chromosome systems or new sex-determining systems (table 2). Namely, certain platyfish (*Xiphophorus maculatus*) populations possess multiple sex chromosomes (X, Y, W; [20]), where Y is dominant over X, and W over Y, so that YY- and XY-individuals develop into males, while XW-, XX- and WY-individuals become females [20,21]. Pure WY versus XY populations had been described by Kallman [20], who also showed that the Y that co-occurs with the W, is homologous to the Y, found in the northern populations with the X, which therefore cannot be deemed Z. Whether this system stems from secondary contacts of incipient species and hybridization still remains unexplored (M. Schartl 2020, personal communication) but it could explain the occurrence of multiple sex chromosomes.

**Table 2.** Expected sexual genotypes and phenotypes in the F_1_ of interspecies crosses at hybridization of an XX/XY and a ZZ/ZW sex determination system, with dominant Y or W versus recessive y or w. While all ZY/Zy genotypes, irrespective of the dominance of the Y, presumably develop into males, all XW/Xw probably become female, whereas XZ phenotypes are hardly predictable, as they depend on the unknown XZ dominance/recessiveness, which may cause ♂ male, ♀ female or ⚥ intersex F_1_-phenotypes.

parents, genotype, phenotype	XY, dominant Y, ♂	XY, recessive y, ♂	any XY, ♂	any XY, ♂	XX ♀
ZW, dominant W, ♀	ZY: presumably ♂	Zy: presumably ♂	XW: presumably ♀	XZ: ♂,⚥,♀	—
ZW, recessive w, ♀	ZY: presumably ♂	Zy: presumably ♂	Xw: presumably ♀	XZ: ♂,⚥,♀	—
ZZ, males, ♂	—	—	—	—	XZ: ♂,⚥,♀

Multiple different sex chromosomes of questionable hybrid origin are also known in anurans. Roco *et al.* [131] showed the coexistence of three sex chromosomes (Z, Y, W) in the clawed frog, *Xenopus tropicalis*, in which no master sex determination gene is known [132]. In laboratory triploids, ZZW genotypes developed as females, but YWW into males, showing the Y is a much stronger male determiner than the Z; while the Z of *X. tropicalis* can determine maleness only in the absence of W [131]. Importantly, commenting on the relative ‘strength' of sex chromosomes, Schartl [133] concluded that this hierarchy in multiple sex-chromosome systems is context-dependent and can vary in different organisms. Recently, nucleotide polymorphisms of expressed transcripts suggested genetic degeneration on the W chromosome, emergence of a new Y chromosome from an ancestral Z chromosome, and natural co-occurrence of the W, Z and Y chromosomes in the same *X. tropicalis* population [134]. Again, a hybrid origin seems likely but is pending confirmation.

Few if any empirical data are available for hybridization of female (ZZ/ZW) and male (XX/XY) heterogametic systems with dominant versus recessive sex chromosomes; table 2 shows the assumed phenotypes under such conditions. Importantly, while all ZY-genotypes may develop as males and XW into females, irrespective of the dominance, XZ phenotypes are hardly predictable, since they depend on the unknown XZ dominance/recessiveness, which may cause male, intersex or female F_1_-phenotypes (table 2).

In Tiliapinae fish, male-heterogamety (XY) on linkage group 1 (LG1) coexists with a female-heterogametic system (ZW) on LG3, sometimes within the same species or populations (e.g. *Oreochromis aureus, O. mossambicus*; [22,23]), where W is dominant over Y, resulting in ZWXY females. Also, in Haplochrominae, a male-heterogamety (XY) on LG7 co-occurs with female-heterogamety (ZW) on LG5, intraspecifically or in populations (e.g. *Metriaclima pyrsonotus* [24]). Again, W dominates over Y, causing ZWXY to be females. The latter authors speculate that interspecific hybrids with different sex-determining systems may produce intersexes with reduced viability or fertility, directly contributing to postzygotic isolation [24]. This suggests that even in early stages of divergence, undifferentiated, in this case non-homologous, sex chromosomes may over-proportionately contribute to the onset of emerging reproductive isolation [135].

Another well-examined teleost example involving, however, heteromorphic sex chromosomes under relatively early divergence, comprises the Central American mosquito fish (*Gambusia holbrooki, G. affinis*), with a divergence time of ca. 2–7 Ma ([31] and citations therein). Here, the heteromorphic ZW sex chromosomes of *G. affinis* females and the homomorphic XY of *G. holbrooki* males present different linkage groups and evolved independently from separate autosomes. In interspecific laboratory hybrids, the Y is dominant over the W chromosome, and X is dominant over Z, in agreement with nonlinear gene flow in a hybrid zone between both species [136].

Hybridization and introgression thus seem to lead to sex chromosome interactions in hardly predictable dominance hierarchies, which either cause ‘evolutionary melting pots' or ‘Darwinian laboratories' with multiple contacts and interactions [137], containing multiple sex loci and/or chromosomes and hypothetically may drive diversification and potentially reinforce the speciation process [135]. More generally, sex-biased introgression and recombination may lead to sex-specific consequences of hybridization and thereby fuel speciation [138].

### (b) Sex chromosomes of hybrids in early stages of divergence: hybrid origin of sex chromosomes and evolution of new sex determination systems

While the systems described above (§2(a)) exemplify that genetic and thus evolutionary interactions by hybridization between incipient or even further separated species may result in hardly predictable outcomes, they nevertheless demonstrate considerable evolutionary impact of sex chromosomes during early divergence. Their introgression may even lead to the establishment of new sex chromosomes and thus sex determination systems. A well-characterized example from teleosts is the Y chromosome in the stickleback, *Pungitius pungitius*. This Y arose by introgression from *P. sinensis* [139], although current hybrid F_1_-males are sterile, females are fertile [140], suggesting that the Y-introgression happened in an early/-ier stage of divergence [139].

An intensely studied anuran hybrid sex chromosome system is that of the Japanese frog *Glandirana* (previously *Rana*) *rugosa*, with five genetic lineages. The West-Japan and East-Japan lineages feature undifferentiated, yet unidentified XX/XY-chromosomes, while the eastern XY-group shows differentiated male heterogamety of chromosome 7. This chromosome bears a ZW sex determination system in northwestern Japan, while a Neo-ZW system occurs in western Central Japan [32,141,142]. The Neo-ZW group, which has a different origin from the ZW-group, shares mitochondrial haplotypes with the geographically proximate XY-group. Nuclear single nucleotide polymorphisms (SNPs) showed the Neo-ZW2 genome to share alleles with the XY-group and partly the Neo-ZW1 group, indicating a hybrid origin of Neo-ZW2. Its sex-linked SNPs on the W stemmed mostly from X chromosomes (XY-group), while alleles on the Z originated from the Z (Neo-ZW1) as well as from Y chromosomes (XY group), suggesting that hybridization of two opposite sex-chromosome systems led to a female heterogametic system by recycling the existing X chromosomes into new W chromosomes. Thus, a new sex-chromosome system evolved by reusing genomic material from ancestral sex chromosomes [33,143]. Populations of *G. rugosa* at the SW-edge of the Neo-ZW group exhibit homomorphic XY-sex chromosomes, but shared mitochondrial haplotypes with the heteromorphic XY-group to the east of its range. Ogata *et al.* [34] concluded that the heteromorphic sex chromosome systems independently reversed back to or were turned over to a homomorphic system at the edges of the Neo-ZW group through hybridization with the West-Japan group, bearing homomorphic sex chromosomes.

Taken together, in relatively earlier stages of divergence, hybridization and introgression of sex chromosomes into foreign gene pools may even lead to the evolution of intermediate or new multilocus sex determination systems. From the examples at hand, this seems much easier in closely related species with undifferentiated sex chromosomes than in more diverged lineages with differentiated sex chromosomes (table 1; cf. [144]). When closely related species differ in their sex determination systems, the outcomes might be more complex than in cases with the same or similar sex determination systems (table 2).

## 3. The ‘extended speciation continuum'

### (a) A new term

Historically, the botanist Alfred Ernst [145] noted that the divergence between parental species predetermines the type of gametogenesis in hybrids—which supposedly follows a continuum from sexual reproduction—when closely related lineages hybridize, through obligately ‘asexual’ hybrid seed production at intermediately distant species, to purely vegetative reproduction in hybrids of distant parents. Focusing on vertebrates, Wetherington *et al.* [146] considered a similar concept, which later was developed by Moritz *et al.* [147] into the ‘balance hypothesis’. It states that the formation of ‘asexually' reproducing hybrids (box 1) is particularly likely when the genetic divergence between parental genomes is large enough to distort hybrid gametogenesis towards producing a high proportion of unreduced gametes, but not too large to significantly affect hybrid viability or fertility. Discussing the balance hypothesis, Stöck *et al.* ([148], supported by [149,150]), also emphasized that ‘asexual' vertebrates are very rarely formed (e.g. 0.5% of reptile species [39,151,152]) since both sufficient divergence and generally complex genetic preconditions are necessary to naturally produce viable and fertile clonal genomes and phenotypes (‘rare formation hypothesis' [148]).

However, once a window of favourable genetic divergences among hybridizing species occurs, the stage is temporally set for specific combinations of their genomes, potentially allowing repeated origins of natural ‘asexual' lineages. These in turn may promote the formation of allopolyploid lineages/species, either immediately or by incorporation of additional genomes upon fertilization of their unreduced gametes (i.e. the ‘genome addition hypothesis', e.g. [35,153]; §3b). Such shifts in hybrid reproduction [46,154] as well as the triggers for allopolyploidization [155,156] have traditionally been examined separately from classical research on speciation, but as we would like to point out, there is a great overlap between both phenomena.

At the molecular level, the mechanisms underlying hybrid sterility and hybrid ‘asexuality' remain elusive but several independently proposed concepts share interesting parallels. For example, Moritz *et al.* [147] proposed that gametogenic aberrations leading to hybrid asexuality arise as a consequence of accumulated gene-to-gene incompatibilities between hybridizing genomes, which conceptually matches the Dobzhansky–Muller genic view on speciation. De Storme & Mason [157] rather proposed that unreduced gametes may be formed in response to decreased homology, preventing proper pairing of orthologous chromosomes, which is analogous to Bateson's [88] non-genic model, currently considered in chromosomal speciation models [158]. Alternatively, Carman [159] suggested that gametogenesis in ‘asexuals' is a consequence of a hampered cross-talk between diverged regulatory programs, combined by hybridization, which exemplifies the important role of postzygotic trans-regulatory incompatibility, recently also considered in speciation research (e.g. [92]).

Hybrid sterility and inviability on the one hand, and a shift in hybrid reproduction to clonality on the other, may both be considered as forms of (partial) postzygotic isolation, evolving along the speciation continuum [38], because the production of clonal gametes by hybrids also reduces the frequency of interspecific introgression by backcrossing into the parental sexual gene pool. As discussed by Janko *et al*. [38], hybrid clonality could thereby contribute to speciation (table 1) before the parental lineages reach complete reproductive incompatibility.

Thus, a century after the seminal works by Bateson [88] and Ernst [145], it appears that the research in the fields of speciation and on hybrid clonal, hemiclonal, meroclonal (‘asexual’) and allopolyploid vertebrates would greatly benefit from greater synergy. To provide a framework for such a synergy and to link the evolution of hemiclonal, clonal or meroclonal ‘asexual’ inheritance mechanisms in allodiploid and allopolyploid species to the concept of the ‘speciation continuum’, we here coin the term ‘extended speciation continuum’.

This new term frames three conceptual steps: profound divergence [147] between two lineages (i) first causes pre-meiotic or meiotic, potentially sex-specific, intrinsic hybrid incompatibilities in gametogenesis ([101, cf. [38]), and (ii) leads to increased potential production of unreduced gametes (e.g. by emergence of endo-duplication) that may rarely either directly lead to the establishment of an ‘asexual' allodiploid lineage/species and/or (iii) at the same time strongly increase the chances of producing unbalanced, meroclonal triploids or directly (or via this ‘triploid bridge' [160]) evolve balanced allotetraploids (cf. [35]). While we develop the concept for vertebrates, future research should evaluate its relevance beyond this group.

### (b) Cytological mechanisms of ‘asexual' reproduction of hybrid vertebrates and link to polyploidy

With few potential exceptions ([75], box 1), all hemi-, mero- and clonally (asexually) reproducing vertebrates are of hybrid origin [144,161], and hemiclonally or clonally reproducing F_1_ progenies have also been obtained upon experimental crossing of certain sexual species ([162] and citations therein). Hybridization thus may affect pre-meiotic processes and/or hybrid meiosis, leading to the production of unreduced gametes with hemiclonal or clonal transmission of (at least parts of) the hybrids' genome [49,163,164]. These forms of ‘asexuality' (*sensu lato*, box 1) in vertebrates, are cytologically characterized by a wide spectrum of gametogenetic mechanisms that range from completely ameiotic processes (apomixis), via hemiclonal mechanisms (classical hybridogenesis) to those involving more or less aberrant meiotic divisions (automixis [165–167], box 1 and figure 1). One gametogenic pathway commonly evolved by ‘asexual' vertebrates is premeiotic endoreplication (figure 1)*,* during which the proliferating germ cells auto-duplicate their chromosome sets, so that identical homozygous copies pair during the subsequent meiotic division, which results in unreduced gametes and a lack of variability among offspring [62,69].

The production of unreduced gametes may consequently pave evolutionary pathways to animal polyploidy, leading to triploid hybrids and then, by further genome addition, to allotetraploids, e.g. by the so-called ‘triploid bridge' ([160,168]; citations in [153]). It has also been proposed that clonal reproduction may facilitate initial establishment of new rare polyploids [35,153,169,170], which may become instantly reproductively isolated from their diploid ancestors and avoid back-crossing producing triploid or aneuploid, potentially infertile progeny. However, empirical data from plants [171] and animals show many exceptions of fertile triploids [50,172,173]. In their balance hypothesis, Moritz *et al.* [147] had also proposed that incorporation of additional genomes into a diploid ‘asexual' hybrid would affect fecundity and viability of allopolyploids by shifts in genome dosages in the hybrids. Such ploidy shifts may cause dosage imbalances between the gene products, potentially causing ‘asexuality'. Indeed, while many triploid hybrid vertebrates with ‘imbalanced’ genomes (e.g. AA'B or AB'B genome-types) usually reproduce by clonal or meroclonal (i.e. ‘partially clonal', box 1) reproductive modes [46], polyploids with ‘balanced' genome configurations, like AA'BB' tetraploids, often reproduce meiotically (e.g. [35,174]), i.e. possibly even facilitating the formation of novel tetraploid species [175]. This suggests that genomic imbalance and divergence are causal for maintenance of clonal reproductive modes [35,147].

Cytogenetically, one may think of these phenomena as follows: Under a certain divergence of hybridizing lineages (cf. [147]), multivalents and thus mis-segregation and chromosome rearrangements during meiosis are expected, posing obstacles to polyploid evolution owing to resulting aneuploidy [35,176,177]. By contrast, fewer inter-lineage multivalents (i.e. of orthologous) may occur when hybridizing lineages exhibit an even greater divergence and genome differentiation [176], i.e. when orthologous chromosomes of the parental lineages no longer match (find) chromosomes in hybrid meiosis, so that new allodiploid ([35,149,157] and citations therein) and especially allopolyploid hybrid lineages [178] may evolve immediately. Indeed, genetic divergence is greater for parents of allopolyploid than of homoploid plant hybrids [179]. Production and/or occasional fertilization of unreduced gametes owing to disturbed premeiotic or meiotic processes in hybrids offers several, in part identical pathways to the evolution of allopolyploid taxa [35,153,179], another evolutionary pathway to overcome hybrid infertility (table 1).

**Figure 2.**
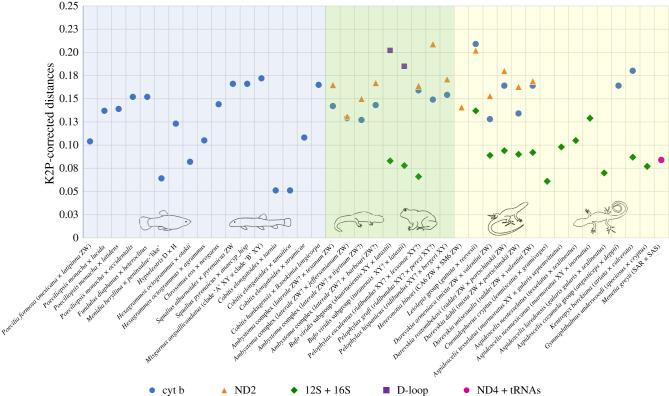
Distribution of the K2P-corrected distances (box 1) between parental taxa of ‘asexual' hybrids in teleost fish, amphibians and reptiles, calculated from different available mitochondrial DNA sequence data. Sequences of cytochrome b (cyt b), NADH dehydrogenase subunit 2 (ND2), NADH dehydrogenase subunit 4 and adjacent tRNAs (ND4 + tRNAs), 12S and 16S rDNA (12S + 16S) and the mitochondrial D-loop (D-loop) were analysed; for details on species names: electronic supplementary material, table S1; for sex determination of parental species: electronic supplementary material, table S2; for data and methodology: electronic supplementary material, file S2. Abbreviations: XY, parental species is male heterogametic XX/XY; ZW, parental species is female heterogametic ZZ/ZW; species names without XY or ZW addition, sex determination in parental species is unknown; ? after XY or ZW indicates that sex determination was inferred (e.g. from crosses or based on apparent evolutionary conservation in the complex).

At least in vertebrates, natural allodiploid and allopolyploid, hemiclonally or clonally reproducing taxa, or even allotetraploid meiotic species, arise mostly at relatively similar divergences between their parental lineages (figure 2). Probably as a consequence, also the likelihood of allopolyploid establishment scales with the genetic divergence between hybridizing lineages [179].

Beyond comprising a potential form of reproductive isolation, ‘asexual' reproduction and evolutionary shifts to allopolyploidy can also present ‘evolutionary escape routes' for hybrids from complete sterility. Indeed, interspecific hybridization may induce alterations of gametogenetic pathways, sometimes giving the hybrid a possibility to alleviate the problems of improper orthologous pairing (e.g. inverted meiosis in butterflies [180]). Likewise, clonal gametogenic pathways, as premeiotic endoreplication, may also enable hybrids to successfully pass meiotic checkpoints [66] and to transmit at least parts of their genomes, despite the problems they experience with postzygotic incompatibilities [35]. Processes involving some type of hybrid-origin clonality allow the existence of hybrid vertebrates in the ‘extended speciation continuum’.

### (c) Empirical support for the concept of the ‘extended speciation continuum’

The assumption that ‘asexual' reproduction may arise as a consequence of accumulating incompatibilities was supported by two meta-studies in hybrid lizards [181] and fish [38] that compared the occurrence of reproductive anomalies in hybrids with the genetic divergence of their sexual parental species, approximated by their mtDNA sequence divergence. The genetic divergence between parental species of these parthenogenetic lizards or gynogenetic fish was significantly higher than between species producing viable gonochoristic/sexual hybrids. Species pairs producing ‘asexual' hybrids were also less diverged than those producing sterile fish hybrids [38]. Similarly, in Palearctic green toads (*Bufo* or *Bufotes viridis* subgroup), the parental lineages of diploid sexually reproducing hybrids at secondary contact zones [15,26] are much more closely related than two deeply diverged nuclear clades (6 Ma) that formed the maternal and paternal ancestors of all meroclonal allotriploid and meiotic allotetraploid taxa [35].

Of note, the production of ‘asexuals' coincides with the formation of sterile hybrids in certain species/hybrid complexes, like e.g. *Cobitis* loaches [38], killifish, *Fundulus* [182] or medaka, *Oryzias* [183]. Natural hybridization between the loaches *Cobitis elongatoides* and *C. taenia*, diverged approximately 9 Ma, yields sterile diploid males with improper chromosome pairing and bivalent formation during the first meiotic division. In diploid hybrid females, gonial cells undergo premeiotic endo-duplication of chromosomes, form bivalents and clonal progeny (figure 1) [38,66]. Hence, both reproductive isolating mechanisms (hybrid sterility and ‘asexuality') may occur simultaneously and some ‘asexual' pathways may not only serve as reproductive barrier but also as at least temporal ‘remedy' preventing sterility.

In addition, we have compiled or calculated the K2P-corrected distances between parental taxa of 41 ‘asexual' hybrids in fish, amphibians and reptiles, analysed from available mitochondrial DNA sequence data (electronic supplementary material, table S1, files S2 and S3). Parental K2P-distance data for lineages of 17 teleost fish, 9 amphibians and 15 reptiles (figure 2) show them all to be greater than approximately 5% and to reach up to approximately 22%. While our data can only be a rough approximation, and part of the observed variation stems from different mitochondrial markers (figure 2), they show that divergences between parental lineages are larger than intraspecific mitochondrial variation in gonochoristic taxa, which typically reach K2P distances of approximately 1–4% in fish (e.g. [184–186]), approximately 1–5% in amphibians (e.g. [187–191]) and approximately 1–3% in reptiles (e.g. [189,191]). Our data suggest that a genetic distance exceeding (most) intraspecific levels presents a major precondition to evolve a natural hybrid ‘asexual' vertebrate.

## 4. Sex chromosomes in hybrids in the extended speciation continuum

### (a) Sex-specific differences of cytogenetic mechanisms, gametogenesis and reproductive modes of hybrid clonal, hemiclonal and meroclonal vertebrates

There is another important aspect of the evolution of ‘asexual’ and several allopolyploid hybrids, which has an apparent analogy to the accumulation of postzygotic reproductive incompatibilities, i.e. the tendency to arise asymmetrically in both sexes. In particular, most ‘asexual' vertebrates exhibit strongly female-biased sex ratios, which is why they have also been referred to as ‘unisexual' or ‘all-female' species [49,164,192,193].

Such a female bias might result from the simple fact that (hemi-)clonal males cannot generate progeny on their own, since their reproduction requires ova; even in cases like androgenesis [194,195], where clonal sperm replaces egg nuclei from related females. This reliance on eggs could explain why hybrid males are often absent in ‘asexual' vertebrate taxa, even if they would be able to produce fertile (hemi-)clonal gametes.

However, there might be more fundamental differences between male and female hybrids in terms of their ability to undergo ‘asexual' gametogenesis. Although studies that compared sex-specific gametogenesis in ‘asexual' vertebrate complexes are scarce, they consistently suggest that hybrid females may reproduce ‘asexually', while males often cannot generate functional sperm [66,196–198]. For instance, research in loaches refers to the basis for different sex-specific outcomes. Hybrid males faced problems with pairing of homeologous/orthologous chromosomes and thus failed to pass meiotic checkpoints. By contrast, hybrid females of unknown genetic sex pre-meiotically endo-reduplicated their chromosomes in the oogonia and formed bivalents, formally recombining between self-duplicated sister chromosomes (auto-copies), which allowed successful accomplishment of oogenesis but yielded no variability among offspring (figure 1). Thus, despite completing the meiotic divisions, females reproduced clonally, while males were sterile [66,198,199]. Another type of asymmetries has been documented in medaka fish *(Oryzias latipes *x* O. curvinotus)*, in which female hybrids yielded clonal ova by premeiotic endoreplication, while hybrid males skipped meiosis and generated a single unreduced diploid spermatozoid from each spermatogonium [183,200].

Differences between sexes exist also in ‘asexuals'' with genome elimination. For instance, in hybridogenetic water frogs (*Pelophylax esculentus*; see below), male and female hybrids typically eliminate the L(*lessonae*)-genome and produce hemiclonal gametes with only the R(*ridibundus*)-genome [201] (figure 1). However, some male hybrids produce the ‘opposite type' of gametes by eliminating the R-genome, while females do not show this genome elimination [202–204] (see below). Similarly, triploid hybrid bisexual Batura-toads (*Bufo*(*tes*) *baturae*; see §5c(iii)) exhibit sex-specific differences in elimination of one genome in males and its separate endoreplication in females (figure 1) [50,73].

Differences in gametogenesis and reproductive modes between male and female hybrids of many clonal, hemiclonal and meroclonal taxa may reflect complex patterns and depend, among others, on hybrid's ploidy and genome dosage. In some cases, diploid and triploid hybrids of the same sex that arose from the same parental species may differ in gametogenesis and/or reproductive modes. For instance, all-female diploid *Poeciliopsis monacha-lucida* hybrids, with an estimated divergence between the parental lineages of 5–6 Ma [205,206], are hybridogenetic (figure 1) [163], while all-female triploid *Poeciliopsis* hybrids reproduce clonally by gynogenesis [51]. Inverse patterns were revealed in the *Cobitis hankugensis* × *Iksookimia longicorpa* hybrid complex, with diploid hybrids reproducing gynogenetically and thus clonally, while triploid hybrids eliminate the single genome and do not undergo endoreplication [196].

Crossing experiments in loaches (*Cobitis*, *Misgurnus*), livebearers (*Poeciliopsis*) and whiptail lizards (*Aspidoscelis*) also demonstrated that the origins of female hybrid (asexuality) and male sterility are directly linked to their hybrid origin since both patterns immediately co-occurred in F_1_-hybrids [51,66,162,198,207]. Moreover, when Yoshikawa *et al.* [208] sex-reverted clonal diploid *Misgurnus* female hybrids into males, such sex-reversed males differed from sterile natural male hybrids by producing unreduced spermatozoa via endoreplication. This suggests that ‘asexual’ gametogenesis may depend on genetic rather than phenotypic sex determination (see §5a), making it tempting to speculate that emergence of ‘asexual' vertebrates could be linked to the evolution of sex chromosomes.

### (b) Sex chromosomes, Haldane's rule and Darwin's corollary at the establishment of hybrid clonal, hemiclonal, meroclonal and allopolyploid vertebrates

When the parental species of an ‘asexual' (or allopolyploid) species exhibits genetic sex determination, it can be assumed that at their initial (F_1_) hybridization Haldane's rule [100,209] could play a role. Importantly, most hybrid vertebrates feature homomorphic (presumably also molecularly undifferentiated) sex chromosomes (electronic supplementary material, table S1), and the question is how much Haldane's rule applies to them at all (§1b). However, if applicable, two hypotheses can be established: (i) ‘asexual' hybrids could be expected to evolve more easily in male heterogametic systems (XX/XY), with hybrid XX females being fitter but the heterogametic XY hybrids (males) being less fit, infertile or even absent. (ii) Alternatively, if ‘asexuality' of hybrid females arises similarly to hybrid sterility or inviability as a by-product of gene-to-gene incompatibilities (§3a), we may expect its preferential occurrence in female heterogametic systems (ZZ/ZW), because recessive incompatibilities first appear in heterogametic females (ZW). Premeiotic or meiotic aberrations, enabling the evolution of ‘asexuals’, would thus present Haldane effects. Intriguingly, the absence of ZZ males (predicted to be fitter) could arise owing to their inability to produce offspring on their own or by counterselection through backcrosses with the parental lineages.

To shed some light on these hypotheses and generally to infer whether sex determination systems play a role at the establishment of an ‘asexual' vertebrate complex, we have compiled the available evidence for sex-determining systems of the parental forms (electronic supplementary material, tables S1 and S2). Assuming that ‘asexuals', which share their parental genomes and just differ by ploidy and quantitative composition (e.g. AB, ABB or AAB), have a common hybrid origin (AB), out of 144 ‘asexual' vertebrate forms, we have chosen 52 complexes (with ancestry information: electronic supplementary material, table S1) that may be traceable to a single separate hybridization event. In 36 cases, out of these 52 complexes, we have no information about parental sex chromosomes/sex determination. In five ‘asexual' complexes, the information about genetic sex is available for only one parental species (2 ZZ/ZW, 3 XX/XY), and from eight ‘asexual' complexes sexual genotypes are known from both parents: 5 with a ZZ/ZW, and 3 XX/XY. Polyploid complexes with multiple (3 or 4) genome donors come exclusively from 3 female heterogametic (ZW) systems. Taken together, among 52 ‘asexual' taxa with known ancestry, for the vast majority of 36, information on sex chromosomes is entirely missing, 10 parental species possess ZW and 6 have XY sex determination systems. This suggests that it could be easier to evolve an ‘asexual vertebrate' in a female heterogametic system (hypothesis ii).

Other reasons underlying the different reproductive capacities of ‘asexual’ F_1_-females and their F_1_-brothers (§4a) at the basal hybridization of an ‘asexual' complex, however, may not be caused by genetic sex determination (only). For instance, Darwin's corollary [103,210] refers to asymmetric fitness in hybrids of reciprocal crosses [111] and Bateson-Dobzhansky–Muller-interactions between autosomal and uniparentally inherited factors, like cytoplasmic elements, maternal transcripts or sex chromosomes in heterogametic hybrids, which depend on the direction of hybridization, thus contributing to asymmetric reproductive isolation between parental lineages. This implies that randomness (i.e. which species is by chance the maternal and which is the paternal ancestor) regarding the direction of initial crosses could also be causal of whether this F_1_ may or may not give rise to a unisexual or allopolyploid lineage. Indeed, the maternal (mitochondrial) ancestors of multiple allopolyploid green toads stem always from the same clades [35], supporting such asymmetry.

A related hypothesis, testable in longer term, is whether hybrid vertebrate complexes with female-biased sex ratios (all-female species) may evolve owing to (or be influenced by) the dominance hierarchy of different (homologous or non-homologous) sex-determining loci of the parental species, e.g. similar to the sex determination systems in platyfish or some cichlids (see §2a).

### (c) Evolutionary expectations for sex chromosomes in polyploids

Except for some of the lizards, most hybrid-origin ‘asexual’ and allopolyploid vertebrates (see also §5d) feature undifferentiated sex chromosomes. This fits theoretical assumptions about the evolution of polyploids and sex chromosomes in general as Muller [211] attributed the rarity of polyploid animals to the disruption of sex determination under polyploidization. Duplication of degenerated sex chromosomes may imbalance sex versus autosomal gene expression [212], implying the rarity of polyploid animals with degenerate Y (or W). Therefore, Otto & Whitton [213] assumed polyploids to occur in animals with: (a) ‘asexual’ and hermaphroditic reproduction, (b) sex determination based on a Y-linked sex determiner rather than an X : A ratio, and (c) non-degenerate sex chromosomes and absence of dosage compensation (e.g. amphibians). Mable [214] and later similarly Wertheim *et al.* [215] excluded a single common explanation for the relative rarity of polyploid animals compared to plants. Using phylogenetic analyses, Evans *et al.* [216] concluded that soon after inferred sex chromosome turnovers in the amphibian phylogeny, polyploidization might evolve more easily and thus more frequently.

Muller [211] drew his conclusions from research on fruit flies, *Drosophila*, in which the X : A(=autosomes)-ratio is disrupted under polyploidy. Wertheim *et al.* [215] predicted the various sexual phenotypes resulting from polyploidization events under male (XY) or female heterogamety (ZW) of diploid parents with either a dominant male (Y) determiner or a dominant female-determining (W) locus (as well as sex chromosomes to autosomes ratios, unknown to play a sex-determining role in vertebrates). Under a dominant Y, the sex ratio is expected to be biased towards the heterogametic sex so that new tetraploids (XXXY, XXYY, XYYY) individuals will likely develop into males and only XXXX-individuals into females. However, strong sex-ratio selection should quickly restore the balance in natural populations [111,213]. By contrast, in female-heterogametic (ZZ/ZW) systems with a dominant W, where three-quarters of progeny (ZZZW, ZZWW, ZWWW) would be female, sex-ratio selection might be weaker. Polyploids would thus arise more easily in ZW-systems (which is, for example, in accordance with ZW-systems of clawed frogs, *Xenopus*; see §5c(i)) than in XY-systems under dominant drivers [215].

However, Wertheim *et al.* [215] did not discuss polyploid hybrids governed by varying numbers and thus dosages of sex chromosomes (table 3; for example, with a recessive Y: XY = male, XXY = intersex, XXXY = female; or with recessive W: ZW = female, ZZW = intersex, ZZZW = male), hybrids with multiple sex chromosomes resulting from allopolyploidy, or hybrids with more complicated dominance hierarchies (e.g. XZW or YZW triploids; XXZW or XYZW tetraploids etc.; for multiple sex loci in diploid hybrids: see §2a). Clawed frogs, *Xenopus* (see §5c(i)), may even have evolved a new master sex-determining gene in response to allotetraploidization [217], suggesting allopolyploidy may also *de novo*-generate a sex determination system.

**Table 3.** Sex chromosomal genotypes and assumed sexual phenotypes of diploids and polyploids of crosses resulting from XX/XY and ZZ/ZW genotypic ancestors in vertebrates under dominant or recessive Y or W chromosomal sex determination. Symbols: ♂ male, ♀ female, ⚥ intersex and ? unclear.

ploidy	sex chromosomes	dominant W	recessive w	sex chromosomes	dominant Y	recessive y
diploid	ZW	♀	♀	XY	♂	♂
triploid	ZZW	♀	⚥ ?	XXY	♂	⚥ ?
tetraploid	ZZZW	♀	♂	XXXY	♂	♀

The complex implications from §§4a–c suggest that the type of hybrid gametogenesis and the sex-specific differences in many clonal, hemiclonal and meroclonal taxa may not only reflect a combination of particular parental genomes and, possibly, sex determination systems, but also their dosage. Whether and how sex-specific cytogenetic mechanisms and reproductive modes are linked to the sex chromosomal genotypes remains an open question.

## 5. Examples of sex chromosomes in hybrid clonal, hemiclonal, meroclonal (‘asexual’) and meiotic allopolyploid vertebrates

According to Neaves & Baumann [161] female-bias is found in about 80 vertebrates, while some form of hybrid clonality (asexuality) has been confirmed in approximately 140 forms of fish, amphibians and reptiles (electronic supplementary material, table S1). While there are major empirical knowledge gaps, here we provide examples for hybrid diploid/polyploid vertebrate complexes, most of which exhibit clonal, hemiclonal or meroclonal reproduction, and the current level of understanding about their sex chromosomal situations (electronic supplementary material, table S1). We focus on examples from fish, amphibians and reptiles that are relatively well-examined and exhibit a variety of sex-determination systems and reproductive modes.

### (a) Teleost fishes

#### (i) Cobitidae

‘Asexuality' is frequently observed in this teleost family. Spined loaches represent a monophyletic, yet deeply divergent group with multiple independent hybridization events, resulting in more than 20 hybrid combinations varying in ploidy levels and reproductive modes, including both gynogenesis and hybridogenesis [72,218–223]. Hybrid females and males notably differ in their ability to reproduce; while diploid and triploid hybrid males are always sterile [196,224–226], hybrid females maintain fertility and reproduce either via gynogenesis or meiotic hybridogenesis (figure 1) [218,220–223]. Male sterility is evident by aberrant pairing of homeologous chromosomes resulting in the failure of meiosis and formation of aneuploid sperm [66,198]. On the other hand, hybrid females show premeiotic endoreplication of chromosomes, allowing normal pairing and meiotic progression with recombining identical copies of chromosomes (figure 1) [66,68,72,199,221]. In dojo loaches (*Misgurnus anguillicaudatus*), sex reversal of females by hormone treatment revealed that such males were able to produce unreduced spermatozoa via endoreplication like hybrid females [208]. This suggests that clonal gametogenesis is linked to female genetic sex and may depend rather on genotypic than on phenotypic sex. Therefore, the question arises whether the hybrid sex chromosomal configuration contributes to the evolution of ‘asexuality' and/or whether the sex-specific outcomes of inter-lineage hybridizations may be other Haldane-effects (§4b). The results suggest that genetic but not phenotypic sex determination controls the endoreplication ability in diploid hybrids. Male heterogametic sex determination was suggested in both dojo (*Misgurnus*) and spined (*Cobitis*) loaches, with the latter genus putatively possessing multiple sex chromosome systems [227–231]. Nevertheless, these reports for *Cobitis* involved individuals of uncertain genetic composition, with the possibility of their hybrid origin, as they had 49 chromosomes and were sampled from isolated populations [228,229]. In other sexual and hybrid species, the analysis of mitotic and meiotic chromosomes did not reveal any morphological differences between sex chromosomes and autosomes [232–234], requiring genomics to reveal potential sex-linked molecular differences.

#### (ii) Poeciliidae

*Poecilia formosa*, the allodiploid hybrid approximately 100 ka-old all-female Amazon molly, produces clonal gametes by apomixis and reproduces by gynogenesis [64] (figure 1), in a system traceable back to a very few initial hybridization events [148,149]. Cytogenetic methods could not clarify the sex-determining system of its maternal (mitochondrial) ancestor [235], *P. mexicana* [236], while its paternal ancestor, *P. latipinna,* exhibits female heterogamety and heteromorphy [235]. Laboratory hybrids between the ancestral species (*P. mexicana* x *P. latipinna*) showed automictic gametogenesis [237] involving the random fusion of meiotic products after the second meiotic division. Masculinized diploid *P. formosa*, obtained by hormonal treatments [238], were examined regarding their sexual phenotype and behaviour, but whether their spermatogenesis is apomictic, like *P. formosa* oogenesis, has not been examined (M. Schartl 2020, personal communication). Natural triploid *P. formosa* are usually female [239,240], while unusual triploid males, possessing supernumerary microchromosomes, showed aberrant spermatogenesis, resulting in aneuploid sperm [149,241]. Genomics showed that genes that serve organs or processes that are no longer in use in the all-female fish, such as spermatogenesis, male development and meiosis genes, are not corrupted [149]. Genomic approaches should in the longer term also allow identification of the sex chromosomes in *P. mexicana* and their elucidation in the allodiploid *P. formosa*, in which most recent transcriptomic analyses of transcriptional divergence between different clonal lineages suggest that functional *P. formosa* allelic expression patterns do not simply reflect the ancestral situation of an F_1_-hybrid but potentially result from long-term selection of transcriptional fitness [242].

### (b) Amphibia, Urodela

The unisexual *Ambystoma* salamander complex comprises at least 24 hybrid combinations of diploid to pentaploid forms [243], involving nuclear genomes of two to five species [63,243–245].

Mostly triploid hybrid females (e.g. LLJ or JJL) undergo a premeiotic endoreplication (endomitosis) leading to hexaploid oocytes. Meiosis produces triploid oocytes that can be activated by sperm from gonochoristic species [245] (figure 1). Female hybrids obtain (steal) this sperm from five bisexual congeneric species, used only to trigger egg development by gynogenesis (sperm-dependent parthenogenesis), or for incorporation into the zygote to elevate the ploidy level (tetraploid to pentaploid), or to replace one of the female's haploid genomes, a reproductive mode in summary called ‘kleptogenesis' [243] (figure 1).

The complex likely arose from an ancient hybridization event of a female close to *Ambystoma barbouri* (providing its mtDNA [61,246]), and a dated phylogeny based on complete mitochondrial genomes [247] suggested the complex to be *ca* 5 Myr old. None of the unisexuals can be considered hybrids between any contemporary species. Although all unisexual combinations of *Ambystoma* include at least one *A. laterale* (L) genome [192], this does not represent the most ancestral hybrid, since the maternal *A. barbouri* ancestry implies that neither *A. laterale* nor *A. jeffersonianum* could have been the female that gave rise to the complex. Instead, the *A. laterale* genome present in all hybrids, as well as those genomes of all other sperm donors in the complex, are considered to come from males (likely containing a Z-chromosome).

**Figure 3.**
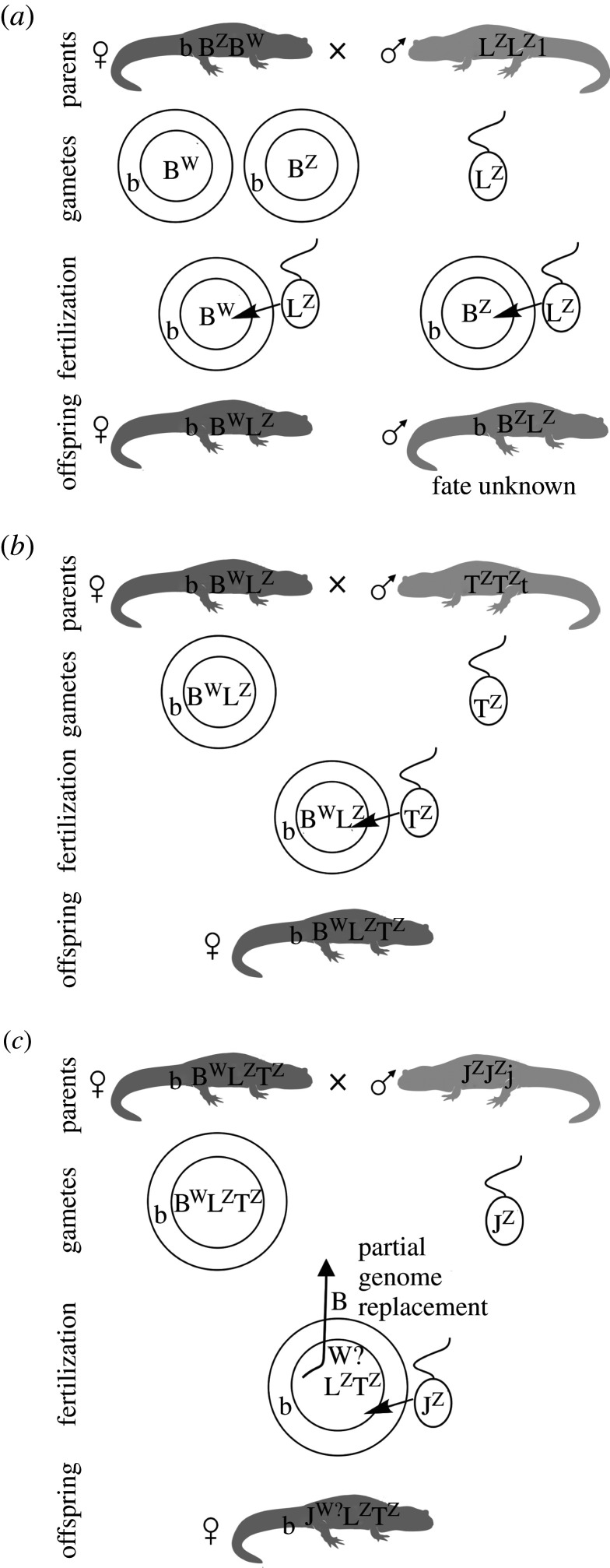
Inferred primary hybridization event at the origin of the kleptogenetic mole salamanders (unisexual *Ambystoma* complex) and hypothetical sex chromosome transmission within this complex. Kleptogenetic unisexual *Ambystoma* (figure 1 and box 1), their gametes and the resulting offspring. (*a*) Mating of a diploid female as the maternal sexual ancestor (*A. barbouri* (B^w^B^z^)) with zW sex chromosomes and a *A. laterale* male (L^z^L^z^) with zz sex chromosomes resulted in a diploid clonal B^w^L^z^ female F_1_-hybrid (left) and possibly a diploid B^z^L^z^ hybrid F_1_-male. (*b*) Cross of the female F_1_-hybrid (B^w^L^z^) with an *A. texanum* (L^z^L^z^) male, sperm incorporation and thus ploidy elevation result in a triploid B^w^L^z^T^z^-female. (*c*) Kleptogenetic reproduction of a triploid (unisexual) B^w^L^z^T^z^-female and an *A. jeffersonianum* (J^z^J^z^) male, resulting in the replacement of the *A. barbouri* (B) genome by a paternal J-genome. The female-determining factor on the W chromosome of *A. barbouri* is hypothetically translocated (possibly by intergenomic recombination, as well known in the complex; [249]) to the J-genome and thus might have caused the emergence of J^w^L^z^T^z^ females. W is the inferred dominant female-determining factor; z indicates recessive male-determining factors; b, j, l, t symbolize *A. barbouri*, *A. jeffersonianum*, *A. laterale* and *A. texanum* mitochondrial DNAs, respectively; silhouettes symbolize *A. jeffersonianum*: dark grey; *A. laterale*: light grey; their diploid, triploid and tetraploid hybrids: intermediate grey. Drawn according to the discussion in Robertson *et al*. [250].

Sessions [248] cytogenetically identified a ZZ/ZW sex chromosome heteromorphism in the diploid nuclear *A. laterale* (LL), and concluded that its Z (L^z^) formed a diploid ancestral hybrid female (J^w^L^z^). The genome of *A. jeffersonianum* including its dominant W (J^w^) appeared thus important to maintain all-female clones, and explained female triploids as J^w^J^z^L^z^ and J^w^L^z^L^z^-genotypes [248]. This explanation, however, is in conflict with the later-identified maternal ancestry by *A. barbouri* that provided its mtDNA [61,246], and, if so, should have also contributed a W-chromosome (B^w^ in figure 3) to the F_1_-hybrid (e.g. B^w^L^z^). Since male sperm donors probably always add Z-chromosomes to the complex that are also considered to have replaced the ancestral nuclear *A. barbouri* genome [251], and thus its W, it remains unknown how a female condition could have evolved or be maintained in the complex. Robertson *et al.* [250] hypothesized that inter-genomic chromosome recombination [249] and translocations [252], which demonstrate that crossovers have occurred between homeologous chromosomes, and not only the sister (endoduplicated) chromosomes, could also have affected the sex chromosomes, and a translocated female, perhaps *A. barbouri* (W)-locus on an *A. laterale* chromosome, might thus explain the femaleness of the kleptogens [250]. Owing to the enormous genome size, genetic information on the sex chromosomes is still missing in the complex but by using genomic approaches female heterogamety (ZW) has also been shown in other *Ambystoma* [253], and generally, a dominant W could sufficiently explain the overwhelming unisexuality in the complex. However, a very few ‘unisexual' males (11 of 962 unisexuals) have been found in the complex; discussed and male meiotic figures provided by Bogart [246]. Molecular details of the sex chromosome evolution, function and interactions remain to be elucidated in the unisexual *Ambystoma* complex.

### (c) Amphibia, Anura

Polyploidy evolved frequently in Amphibia (e.g. [156,214]) with 50 anuran and six salamander species [216], including many allopolyploids. All known polyploid anurans feature poorly differentiated (homomorphic) sex chromosomes. Here, we focus on an example of a polyploid complex of allopolyploids with even ploidies (*Xenopus*), a hybridogenetic complex involving triploids (*Pelophylax*) and on diploid and tetraploid meiotic but meroclonal triploid hybrids (*Bufo*).

#### (i) Pipidae

Clawed frogs (*Xenopus*) comprise the largest ploidy-range known in an anuran radiation, reaching from diploid to do-decaploid (12n), all of which appear to be of hybrid origin [254]. Diploid *X. tropicalis* features W, Z and Y sex chromosomes (discussed §2(a)). Subgenome evolution in allopolyploids has only recently been studied in *Xenopus laevis* [255,256]. Its female-determining gene *Dm-W* is situated on the undifferentiated chromosome (2 L) and presents the only well-characterized anuran master sex determiner, a paralog of *Dmrt1* [142,257], and arose after (and perhaps in response to) tetraploidization [217,258,259]. It is also found in some related *Xenopus* [258–260] but not in the entire radiation. Allotetraploid *Xenopus borealis* lost *Dm-W* and evolved new sex chromosomes on chromosome 8 L (chr8 [134,261]). Song *et al.* [261] summarized the variance in recombination suppression around the sex-linked portions to be very small in *X. tropicalis* and *X. laevis* but almost half the sex chromosomes in *X. borealis*, the other half presenting a pseudoautosomal region [260]. Although all polyploids are of hybrid origin, to our knowledge, no clonal or hemiclonal forms are known in *Xenopus* but only gonochoristic meiotic lineages with even ploidies. The elucidation of sex evolution and its role in this anuran radiation will continue to provide major insights into the links between sex determination and allopolyploidy in vertebrates.

#### (ii) Ranidae

**Figure 4.**
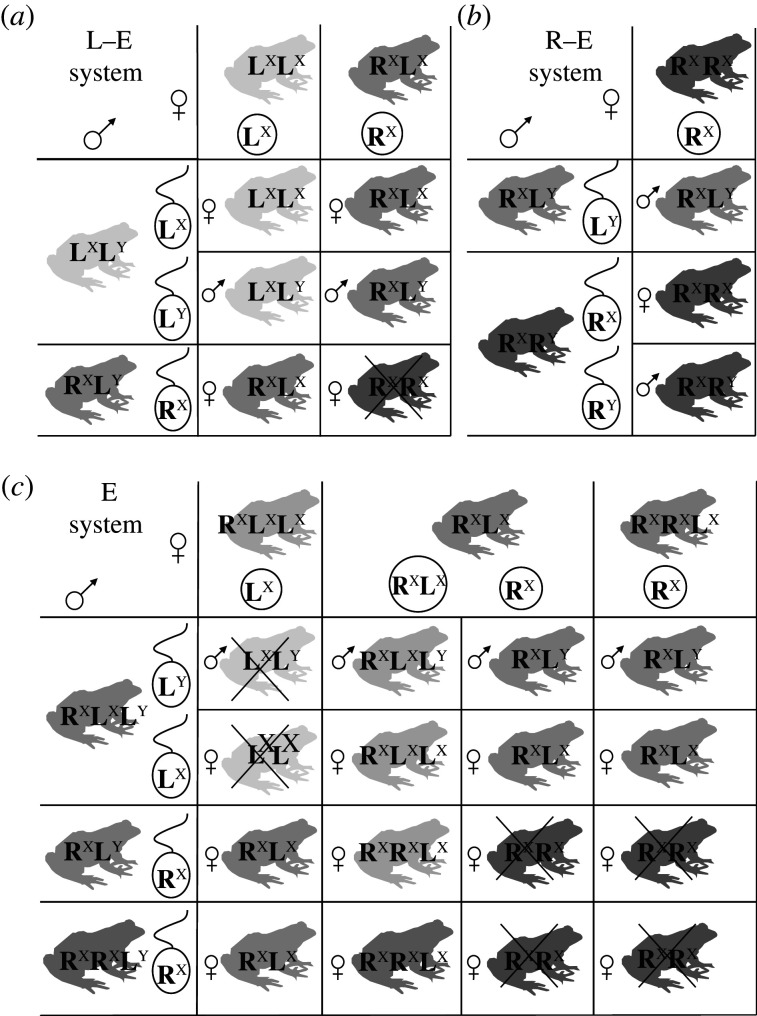
Inferred inheritance of male heterogametic (XY) sex determination loci in three hybridogenetic population systems of Western Palearctic water frogs (*Pelophylax esculentus* complex). Reproduction and inheritance of sex-determining loci in water frogs in three different population systems with diploid and/or triploid hybridogenetic (hemiclonal and meroclonal, box 1) hybrids. (*a*) In the L–E system (gonochoristic *P. lessonae* reproduce with hybridogenetic *P. esculentus*), the Y-factor, restricted to the L-genome, produces female excess among R^x^L^x^-hybrid offspring. R^x^R^x^-offspring typically die before reaching sexual maturity (crossed out). (*b*) In the R–E system (gonochoristic *P. ridibundus* reproduce with hybridogenetic *P. esculentus*), diploid R^x^L^y^ hybrid genotypes are all-male. (*c*) In the E system (‘pure' diploid and triploid hybrid *P. esculentus* hybrids only), the dominant male-determining Y factor supposedly only occurs in males' L-genomes (L^y^). Any resulting non-hybrid offspring (LL, RR) do not survive to sexual maturity. Male RRL offspring (R^x^R^x^L^y^) are usually not formed by the crosses of this population system. Frog silhouettes, their gametes and resulting offspring are shown. *P. lessonae* (LL): light grey; *P. ridibundus* (RR): dark grey; diploid (RL) and triploid hybrids (RRL and RLL): intermediate grey; redrawn from Christiansen [172].

The Western Palearctic water frogs of the *Pelophylax esculentus* (previously *Rana esculenta*) complex include two parental species, *Pelophylax ridibundus* (RR) and *Pelophylax lessonae* (LL), and their natural hybrid forms *P. esculentus*, which are either allodiploid hybridogenetic (RL) or allotriploid (LLR or LRR) (figure 1); other hybridogenetic forms include additional parental species (electronic supplementary material, table S1). A striking feature of *esculentus*-hybrids that distinguishes them from most other clonal and hemiclonal vertebrates is the frequent incidence of males [262]. According to the comprehensive reviews by Günther [262] and Plötner [263], about 15 population systems occur, in which unisexual (either male or female) or bisexual (male and female) diploid and/or triploid *esculentus* hybrids coexist with either parental gonochoristic species. This complex comprises at least *P. lessonae* (five L-e-systems) or *P. ridibundus* (seven R-e-systems) or both (two L-R-e-systems). Uniquely, so-called ‘all-hybrid populations' (e-system) occur, composed of diploid (RL) and triploid (RLL, RRL) *esculentus* hybrids that genetically interact and depend on their specific gamete contributions for successful reproduction, as therein, the parental genotypes *P. lessonae* (LL) and *P. ridibundus* (RR) are absent among adults [59,262]. At least two additional diploid European hybridogenetic forms exist, *Pelophylax grafi* (RG), an allodiploid hybrid between *P. ridibundus* and *Pelophylax perezi* [264], and *Pelophylax hispanicus* (RB), an allodiploid hybrid between *Pelophylax ridibundus* and *Pelophylax bergeri* [265,266]. Importantly, all hybridogens contain at least one *ridibundus* (R)-genome. Various forms of hemiclonal inheritance have been described from allodiploid RL-hybrids, with either L-elimination and clonal inheritance of R or *vice versa* or even diploid RL, LL and RR gametes (figure 1) [60,262,263,267]. Triploid hybrids usually eliminate the genome, which is single (RRL: L; RLL: R), but also produce occasional RL, LL and RR gametes (figure 1) [172]. The karyotypes of the parental *P. ridibundus* and *P. lessonae* can be distinguished by few cytogenetic markers [268] but sex chromosomes were indistinguishable [269]. Like many ranid frogs [270], water frogs have an XX/XY sex determination system. This is suggested mostly from crossing experiments, involving water frogs from many parts of Central and Eastern Europe ([271] and citations therein), by inheritance patterns of allozymes for *P. lessonae* [272], and assumed for diploid hybrid *P. esculentus* [273], but the latter presenting a potential misinterpretation of the hybrid RL-karyotype. In all-hybrid populations, XX/XY-sex determination involves a dominant Y, exclusively on the L-genome [172,201,274], which is either L^X^ or L^Y^, while all R-genomes are R^X^ [59]. Therefore, LLR and LR genotypes can be male (L^X^L^Y^R^X^; L^Y^R^X^) or female (L^X^R^X^; L^X^L^X^R^X^), but most LRR are females [275]. Based on microsatellite analysis of parents and offspring (sexed by dissection) from crossing experiments, Christiansen [172] confirmed sex determination as XX/XY with the Y confined to the L-genome. From crossings, gamete frequencies could be deduced. A model explained genetic interactions of di- and triploid hybrid frogs in self-sustaining populations (figure 4). Both sexes of RLL and RRL produced haploid gametes from the genomes they had twice, while RRL also made 10% LL gametes by automixis. LR frogs showed much variation in their gamete production. In RRL-rich populations, their RL sperm production was high (22%) to explain the observed proportion of RRL males [172]. Populations with biased sex ratio were long known in this complex. Such populations include *P. ridibundus* of both sexes associated with exclusively diploid hybrid males [201] that produce either the L^Y^ genome or the R^X^, leading to the emergence of only hybrid (*esculentus*) males or *P. ridibundus* females after crosses with *P. ridibundus* females [204]. To date, the studies by Christiansen [172,275] appear the most comprehensive ones to include sex chromosome information and sex determination in water frogs. Nevertheless, knowledge on master sex-determining genes, potential intraspecific variation (as observed in other ranid frogs [276]) and on their molecular genetic interactions in the hybrids is lacking.

#### (iii) Bufonidae

In Palearctic green toads, Stöck *et al.* [277,278] have identified secondary contact and hybrid zones in a phylogeographic framework. In diploid/diploid contacts, introgression scales with divergence; i.e. with the degree of speciation [15,16,279]. A range-wide multi-locus phylogeny [35] involved 15 green toad taxa and showed that at least five separate allotriploid and allotetraploid taxa evolved in the Pleistocene. The maternal and paternal ancestors of hybrid polyploids exclusively stem from two deeply diverged (6 Ma, 3.1–9.6 Ma) nuclear clades, with distinctly greater divergence than the parental species of diploid hybrids, found at secondary contact zones. Presumably in all allotriploid forms (electronic supplementary material, table S1), but best examined in Batura toads (*Bufo*(*tes*) *baturae*), two conspecific genomes (NOR+) and a deeply diverged allospecific one (NOR–) are found, suggesting that genomic imbalance and divergence are the reasons for their meroclonal reproductive mode: ‘pre-equalizing hybrid meiosis' (figure 1) [50,73]. The maternal and paternal genome contributions appear asymmetric, with the maternal nuclear (and mitochondrial) genomes of all polyploids constantly stemming from the same clade, and the paternal genome from the other, pointing to a potential role of Darwin's corollary (§4b). Using cytogenetics and inheritance patterns, Stöck *et al.* [280] and Betto-Colliard *et al.* [281] established that diploid and allotetraploid toads reproduce meiotically. At least the imbalanced allotriploid species *B. baturae* reproduces partly clonally [50,73]. Sex chromosomes of diploid toads have been characterized using microsatellites and nuclear sequence markers [282–285], showing that the linkage group, homologous to autosomal LG1 in *X. tropicalis* and harboring *Dmrt1*, is sex-linked in several diploid species of green toads. Male heterogamety (XY) exhibits drastically reduced X–Y recombination in green toads in general, but occasional X–Y recombination occurs on evolutionary time scales [283]. LG1 appears to represent the sex chromosomes in all so far tested diploid green toad species (*Bufo siculus, B. shaartusiensis, B. balearicus, B. turanensis, B. variabilis, B. viridis* and probably *B. boulengeri*). Phylogenetic analyses of a 600 bp fragment of *Dmrt1* furthermore showed that X and Y alleles of this gene cluster by species and not by gametologue. This suggests that XY-sequence similarity stems from occasional XY-recombination involving *Dmrt1*, which preliminarily rejects its role as the master sex determination gene, pending future extension of this evidence to the entire *Dmrt1* gene [285]. The details of sex determination in the allopolyploids have not been examined.

### (d) Reptiles

Approximately 40 species complexes (full list: electronic supplementary material, table S1), i.e. only 0.4% of known squamate reptiles [151,286], are obligately parthenogenetic (box 1), and with few potential exceptions arose via hybridization between sexually reproducing progenitors [46]. Hybrid-origin parthenogenesis is known, for example, in the families Gymnophthalmidae (*Loxopholis,* formerly *Leposoma* [287,288], Gekkonidae (see below), Lacertidae (*Darevskia* [289] and Teiidae (*Aspidoscelis*, *Cnemidophorus*) [70,290,291]). Many parthenogenetic reptile species are clonal hybrid triploids, while tetraploids, with few exceptions [40,292], were only produced by laboratory crosses [291].

#### (i) Teiidae

Parthenogens in *Aspidoscelis* (formerly *Cnemidophorus*, [293]) evolved by hybridization of two diverged mtDNA clades [294]. Gonochoristic *Aspidoscelis* seem to exhibit XY sex determination with slightly heteromorphic sex chromosomes [295]; male and female *de-novo* F_1_-hybrids remain sterile or have unknown fertility [295]. Unisexual *Aspidoscelis* reproduce by premeiotic endomitosis and sister chromatid pairing (figure 1) [69,70]. While natural tetraploids with three parental genomes (trihybrids), resulting from hybridization of triploid lineages with sexual males, are sterile or their fertility remains unknown [296], a self-sustaining 4n lineage was produced in the laboratory [291], raising the even nowadays unresolved question of what constrains development of cascading polyploid series as seen in some invertebrates [41]. That all-female reptiles apparently have evolved in a male heterogametic system appears an exception, since most well-examined cases seem based on ZW-systems with a recessive or dominant W. Initially, the rise of diploid hybrid parthenogenetic lineages [42] would be consistent with the expectation that homogametic XX female hybrids are fitter than male hybrids. Fertilization of parthenogenetic XX females by XY males of a parental or even third species [46,297] elevates these unisexual lineages to triploids [298]. Under dominant male heterogamety, XXY genotypes would be males and possibly also suffer from Haldane-effects (unfit, inviable, infertile), while XXX genotypes would be female and, if so, tri-hybridity of diverged genomes increases the heterozygosity and may even reinforce or ensure clonal oogenesis owing to mismatched chromosomes. Despite the great efforts to reveal the cytogenetic mechanisms of gametogenesis [69] and research on ploidy elevation [291] in *Aspidoscelis*, the elucidation of their sex chromosomal situation remains to be done.

#### (ii) Lacertidae

**Figure 5.**
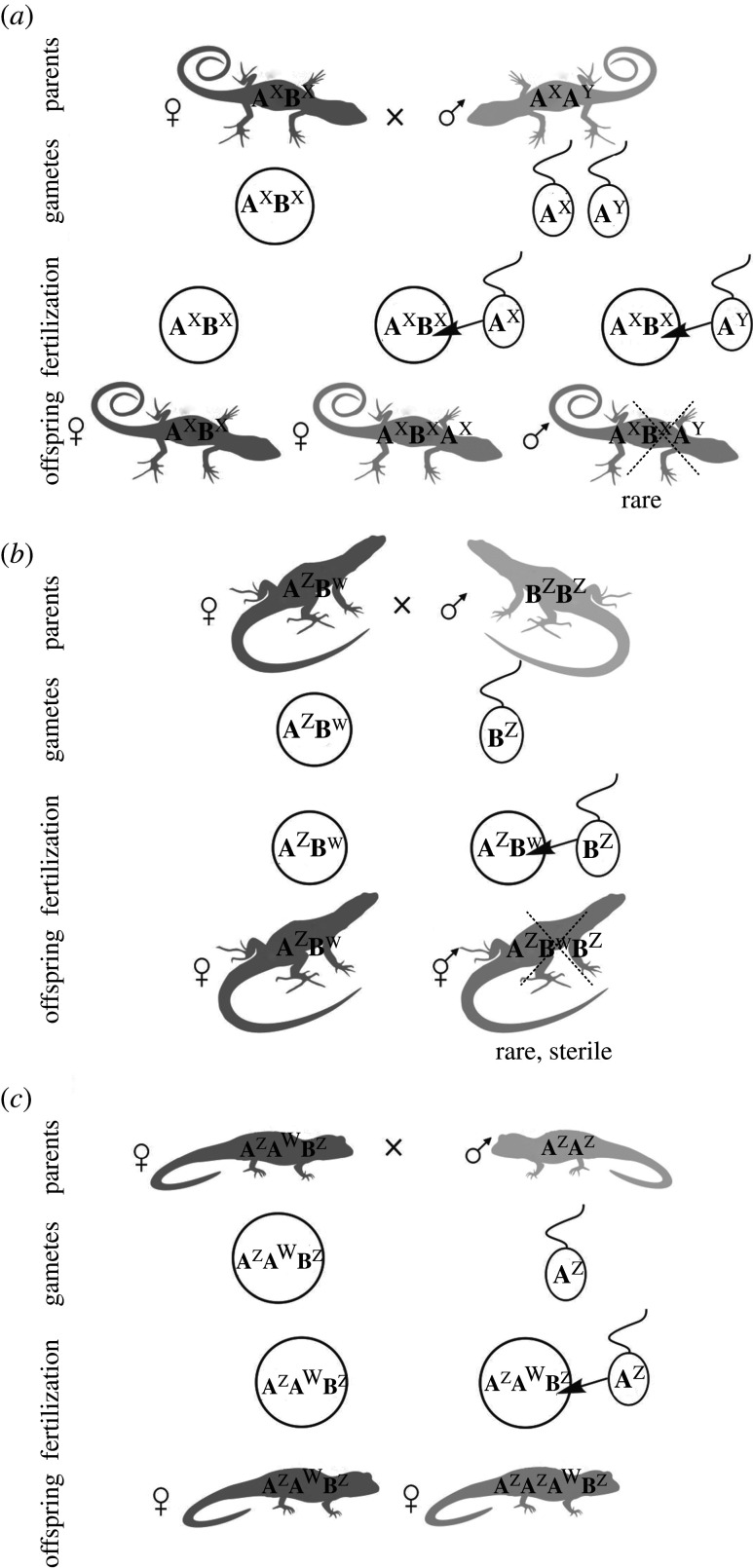
Reproduction and sex determination systems in three parthenogenetic lizards with different sex-determining systems. (*a*) *Aspidoscelis* with male heterogamety (XX/XY): all-female diploid hybrids (AB, left) normally reproduce parthenogenetically; rare fertilization by gonochoristic parental species' males (right) leads to ploidy elevation to a new XXX-triploid parthenogenetic form (X-sperm) but possibly to rare or inviable XXY-males (right, Y-sperm); inferred from Moritz & Bi [41]. (*b*) *Darevskia* with female heterogamety under a recessive w chromosome: occasional fertilization of a diploid parthenogenetic hybrid Zw female by sperm with a dominant male Z factor from a gonochoristic species leads to sterile triploid ZZw intersex genotypes; redrawn from Spangenberg *et al*. [197]. (*c*) *Heteronotia* with female heterogamety under a dominant W chromosome: occasional fertilization of allotriploid parthenogenetic zzW females (left) by sperm with a recessive male z-factor leads to rare tetraploid zzzW genotypes that also develop into females (right); drawn after Moritz [292]. Lizard silhouettes, their gametes and the resulting offspring are shown; arrows indicate occasional fertilization causing ploidy elevation.

The Caucasian parthenogenetic lacertid *Darevskia* present allo-diploid hybrids (figure 1) [299–301], with known hybrid compositions [39,294,302]. Only certain combinations of inter-clade hybridizations of bisexual species (*caucasica* clade and *rudis* clade) led to diploid parthenogenetic lineages, despite numerous records of natural within-clade hybridizations [295]. The ancestral clades show deep divergences (discussed by Avise [46]) and possibly can all be traced back to a few initial hybridization events [39], seemingly supporting the ‘rare formation hypothesis' (see §3(a)). Murphy *et al.* [289] proposed sex chromosomes to play key roles in the formation of unisexual *Darevskia*, which like most lacertid lizards [303] feature female heterogamety (ZW). Murphy *et al.* [289] stated that unisexual *D. dahli* and *D. armeniaca* express the micro-heteromorphic W chromosome from their maternal ancestry, *D. mixta* [304,305], while *D. unisexualis* expresses the derived micro-heteromorphic chromosome from its maternal lineage, *D. raddei* [295,304]). Furthermore, the W chromosome in the maternal gonochoristic *D. raddei* appeared polymorphic while the W chromosome of *D. rostombekowi* is more similar in size and heterochromatin patterns to the paternal ancestor *D. portschinskii* than to the maternal ancestor [295,304]. Likewise, most recently, Spangenberg [306] suggested the recessive w chromosome in unisexual *D. rostombekowi* to be inherited from the maternal ancestor *D. raddei*. Murphy *et al*. [289] further assumed that genes on the highly derived W chromosome might be a prerequisite for unisexuality, as suggested by the sister-relationship of both maternal ancestors. Accordingly, the combination between W-chromosomal genes of the maternal clade (*caucasica*) and Z chromosomal ones from the paternal clade (*rudis*) interrupts normal meiosis and produces unreduced viable eggs. Based on a single parthenogenetic female, Spangenberg *et al.* [197] confirmed synapsis of autosomes during meiotic prophase I, but asynaptic Z and recessive w, and suggested automixis with homeologous autosomes and Zw-sex chromosomes (figure 5), restoring diploidy by central fusion [71] (figure 1). Interestingly, triploid *Darevskia* remain sterile, perhaps because of dosage complications at higher ploidy hybrids, although meiotic instability owing to unpaired chromosomes could also explain the rarity of fertile triploids [152]. In sympatric populations of parthenogenetic *D. unisexualis*—from matings with males of the gonochoristic *D. valentini*—natural triploid hybrids result [197]. A single triploid (*D. unisexualis × D. valentini*) ZZw-male showed distorted synapsis, disturbed meiotic prophase I, passing of meiosis II, but spermatogenesis produced abnormal spermatids [197]. Sexual genotypes and phenotypes in *Darevskia* appear consistent with a recessive w sex determination, in which the number of Z chromosomes in ZZw-triploid hybrids affects femaleness.

#### (iii) Gekkonidae

In Gekkonidae, five all-female species complexes in five different genera are obligate parthenogens [307], *Lepidodactylus* [307,308], *Hemidactylus* [308,309], *Heteronotia* [310], *Hemiphyllodactylus* [311] and *Nactus* [312]. Interestingly, in the molecular phylogeny of squamates (e.g. [313]), all belong to one of two major subclades of Gekkonidae, and all appear to be female heterogametic (ZW). It would be very interesting to elucidate whether their sex chromosomes are homologous. While the knowledge about sex chromosomes in Gekkonidae has strongly increased in the past years [314,315], several reported cases of sex chromosome heteromorphism (e.g. *Lepidodactylus lugubris* [316] that may also have a complex origin according to Trifonov *et al.* [307]; males are infertile: [317], and *Hemidactylus vietnamensis* [309]), may not present sex chromosomes but fixed heterozygosities in certain clonal lineages [318].

Triploid parthenogenetic *Heteronotia binoei* have independent reciprocal hybrid origins from two cytogenetically characterized sexual lineages (CA6, SM6) [292]. While the triploid parthenogenetic form (3N1) has mtDNAs derived from CA6 sexual females, 3N2 parthenogens share mtDNAs with bisexual SM6 [319]. Therefore, some triploids have two CA6 nuclear genomes copies (form A) and others two SM6 nuclear genomes (form BC). The split between the sexual ancestral lineage, which gave rise to multiple hybridizations, was estimated at 5.7–6.5 Ma [320]. Despite the existence of a heteromorphic sex chromosome pair in some diploid populations, parthenogenetic *Heteronotia* have homomorphic sex chromosomes but show different C-banding patterns between Z and W. The W is also cytogenetically polymorphic in several parthenogens [310]. Moritz [292] stated the existence of a dominant W, since ZZW-triploids but also four tetraploid individuals (ZZZW) that arose by fertilization of a parthenogenetic triploid from a sexual species' male [292] were females (figure 5).

## 6. Conclusion

Well-differentiated sex-chromosomes in mammals and birds tend to evolve with unequal rates, potentially causing Haldane-effects in hybrids, presumably owing to relatively well-examined dosage imbalances in the heterogametic hybrid sexes (XY-males, ZW-females). With few exceptions, heteromorphic sex chromosomes in hybrid zones introgress less easily than autosomes into the other species' gene pools. Judged from the limited examples, undifferentiated vertebrate sex chromosomes in earlier stages of divergence, when involved in hybridization and introgression, exhibit a variety of evolutionary outcomes. They may contribute to the emergence of multiple-sex chromosome systems with genetic interactions in hardly predictable dominance hierarchies, where multiple sex loci and/or chromosomes may drive diversification and potentially reinforce the speciation process. Empirical data further suggest that introgression of sex chromosomes under early divergence may not only result in evolutionary genetic interactions (e.g. in hybrid zones) but even lead to the evolution of new sex-determining systems in the affected lineages.

Under greater divergences in the ‘extended speciation continuum', just before most vertebrate hybrids already exhibit complete intrinsic reproductive isolation, a few interspecific hybrids show sex-specific distortion of gametogenesis towards female clonality, which may be caused or influenced by hybrids' genotypic sex. Analysing 41 hybrid ‘asexual' fish (17), amphibian (9) and reptile (15) taxa, we show that K2P-corrected distances, based on different mtDNA fragments of parental species, are larger than approximately 5% reaching up to approximately 22% (figure 2). This supports the hypothesis that ancestral divergence is of major importance in evolving a natural ‘asexual’ vertebrate.

Up to now, the technological limitations in detecting undifferentiated sex chromosomes, sex determination loci and thereby systems in these taxa have caused scarcity of this kind of data for most such vertebrate complexes. Likewise, dominance and recessiveness of sex chromosomes in hybrids remain widely underexplored in many diploid ancestral groups of ‘asexual taxa'.

Although most ‘asexual' vertebrates probably feature genetic sex determination, the evidence (§5), in line with theory (§4c), shows that most ‘asexual' as well as meiotic allopolyploid vertebrates, with the exception of a few lizards, possess undifferentiated sex chromosomes.

The fields of sex chromosomes and sex determination in the ancestral lineages of ‘asexual' as well as some meiotic allopolyploid vertebrate complexes remain widely underexplored (see above; electronic supplementary material, table S1). Hybrid ‘asexual' vertebrates can emerge from parental species with either XY or ZW sex determination systems. However, based on limited data (electronic supplementary material, table S2), it seems more likely to evolve an all-female hybrid form parents with female heterogamety (ZW). Diploid ‘asexual’ *Darevskia* possess a recessive w, and thus rare triploid ZZw genotypes are infertile*.* A dominant W, as inferred in triploid (and rare tetraploid) *Heteronotia*, and triploid to pentaploid *Ambystoma* ensures that sex chromosome dosage increase by Z-chromosome additions under ploidy elevation is possible without compromising the femaleness and thus fertility of these ‘asexuals' (figure 5)*.* From the scarce data (§4b), out of 52 ‘asexual' taxa with known ancestry, information on sex chromosomes is entirely missing for 36. Of the remaining 16, ten known parental species are female heterogametic (ZZ/ZW), whereas only six are male heterogametic (XX/XY), suggesting that it might be easier to evolve an ‘asexual vertebrate' in a ZZ/ZW system (§4b: hypothesis ii). This could mean that Haldane's rule might be relevant to understand ‘asexual' vertebrate evolution, since such ZW females present the heterogametic sex and their premeiotic or meiotic aberrations could be Haldane-effects that promoted the evolution of these ‘asexual' females, while hybrid males got lost over time since either they could not reproduce by themselves or owing to backcrosses.

We further hypothesize that under male heterogamety (XY), the evolution of all-female polyploid taxa may be generally less probable since a dominant Y male determiner (cf. [215]) would not lead to female hybrids but to males only (XXY, XXXY), whereas a recessive y may lead to Xy diploid male hybrids, possibly XXy intersexes and perhaps XXXy females. More complex evolutionary interactions and potential dominance hierarchies in hybrids, resulting from XX/XY and ZZ/ZW parental forms, which generate complex sex chromosomal hybrid polyploid situations (e.g. XWY, XZW, etc.) may lead to individual and hardly predictable outcomes, further contributing to the theoretically [215] and empirically [147] shown examples.

Importantly, molecular information on the sex determination loci and/or master genes is so far only available in very few hybrid allodiploid or allopolyploid systems. Such data as well as mechanistic insights into sex chromosomal evolutionary effects under hybridization may be keys for a full future understanding of the field. Sex chromosome and sex determination research in ‘asexual' and allopolyploid vertebrates in context to speciation appears underexplored and calls for integrative approaches combining rigorous crossing experiments, and the application of cutting-edge techniques reaching from cellular biology, cytogenetics and genomics, to *sexomics* [321], to close these research gaps for a comprehensive understanding of their evolution (see also [286]).

## Data accessibility

Data and materials have been previously published or can be found in the electronic supplemental material.

The data are provided in the electronic supplementary material [322].
